# Modelling the lactate response to short-term all out exercise

**DOI:** 10.1186/1476-5918-6-10

**Published:** 2007-11-09

**Authors:** Ralph Beneke, Masen D Jumah, Renate M Leithäuser

**Affiliations:** 1 Centre for Sports and Exercise Science, Department of Biological Sciences, University of Essex, Wivenhoe Park, Colchester, UK; 2 Helios-Klinikum Berlin-Buch, Hals-Nasen-Ohrenklinik, Berlin, Germany; 3 Biomedical Science, Department of Biological Sciences, University of Essex, Wivenhoe Park, Colchester, UK

## Abstract

**Background:**

The maximum post exercise blood lactate concentration (BLC_max_) has been positively correlated with maximal short-term exercise (MSE) performance. However, the moment when BLC_max _occurs (TBLC_max_) is rather unpredictable and interpretation of BLC response to MSE is therefore difficult.

**Methods:**

We compared a 3- and a 4-parameter model for the analysis of the dynamics of BLC response to MSEs lasting 10 (MSE10) and 30 s (MSE30) in eleven males (24.6 ± 2.3 yrs; 182.4 ± 6.8 cm; 75.1 ± 9.4 kg). The 3-parameter model uses BLC at MSE-start, extra-vascular increase (A) and rate constants of BLC appearance (k_1_) and disappearance (k_2_). The 4-parameter model includes BLC at MSE termination and amplitudes and rate constants of increase (A_1_, y_1_) and decrease (A_2_, y_2_) of post MSE-BLC.

**Results:**

Both models consistently explained 93.69 % or more of the variance of individual BLC responses. Reduction of the number of parameters decreased (p < 0.05) the goodness of the fit in every MSE10 and in 3 MSE30. A (9.1 ± 2.1 vs. 15.3 ± 2.1 mmol l^-1^) and A_1 _(7.1 ± 1.6 vs. 10.9 ± 2.0 mmol l^-1^) were lower (p < 0.05) in MSE10 than in MSE30. k_1 _(0.610 ± 0.119 vs. 0.505 ± 0.107 min^-1^), k_2 _(4.21 10^-2 ^± 1.06 10^-2 ^vs. 2.45 10^-2 ^± 1.04 10^-2 ^min^-1^), and A_2 _(-563.8 ± 370.8 vs. -1412.6 ± 868.8 mmol l^-1^), and y_1 _(0.579 ± 0.137 vs. 0.489 ± 0.076 min^-1^) were higher (p < 0.05) in MSE10 than in MSE30. No corresponding difference in y_2 _(0.41 10^-2 ^± 0.82 10^-2 ^vs. 0.15 10^-2 ^± 0.42 10^-2 ^min^-1^) was found.

**Conclusion:**

The 3-parameter model estimates of lactate appearance and disappearance were sensitive to differences in test duration and support an interrelation between BLC level and halftime of lactate elimination previously found. The 4-parameter model results support the 3-parameter model findings about lactate appearance; however, parameter estimates for lactate disappearance were unrealistic in the 4-parameter model. The 3-parameter model provides useful information about the dynamics of the lactate response to MSE.

## Background

In the early part of the last century, lactate was identified as an indicator of glycolytic activity [1-3]. Soon afterwards, it was observed that blood lactate concentration (BLC) continues to increase for a significant period after termination of maximal short-term high-intensity exercise (MSE) before it starts to decrease [4,5]. The maximum of the post exercise blood lactate concentration (BLC_max_) has been positively correlated with the ability to tolerate high levels of BLC, required to perform well in MSE events [6-8]. An increase in lactate enables calculation of the energy derived from anaerobic metabolism via glycolysis [6,8]. However, the moment when BLC_max _occurs (TBLC_max_) is rather unpredictable and interpretation of BLC response to MSE is difficult.

In the early 1980s, a bi-exponential 4-parameter model was developed to analyse the dynamics of lactate after exercise [9-12]. It describes the post exercise BLC based on two compartments, the working muscle and the non-muscular lactate space, and assumes that the intra-vascular lactate concentration represents the average lactate concentration in the non-muscular space [11]. The two exponential terms describe the post exercise flux of lactate from the muscle into the non-muscular space and the subsequent disappearance of lactate out of the non-muscular space. It includes the BLC at the end of exercise (BLC_end_Ex_), the amplitude (A_1_) and rate constant (y_1_) of the increase and the amplitude (A_2_) and the rate constant (y_2_) of the decrease of the BLC (Eq.1).

BLC(t)=BLCend_Ex+A1⋅(1−e−y1⋅t)+A2⋅(1−e−y2⋅t)
 MathType@MTEF@5@5@+=feaafiart1ev1aaatCvAUfKttLearuWrP9MDH5MBPbIqV92AaeXatLxBI9gBaebbnrfifHhDYfgasaacPC6xNi=xI8qiVKYPFjYdHaVhbbf9v8qqaqFr0xc9vqFj0dXdbba91qpepeI8k8fiI+fsY=rqGqVepae9pg0db9vqaiVgFr0xfr=xfr=xc9adbaqaaeGacaGaaiaabeqaaeqabiWaaaGcbaGaeeOqaiKaeeitaWKaee4qamKaeeikaGIaeeiDaqNaeeykaKIaeyypa0JaeeOqaiKaeeitaWKaee4qam0aaSbaaSqaaiabbwgaLjabb6gaUjabbsgaKjabb+faFjabbweafjabbIha4bqabaGccqGHRaWkcqqGbbqqdaWgaaWcbaGaeeymaedabeaakiabgwSixlabbIcaOiabbgdaXiabgkHiTiabbwgaLnaaCaaaleqabaGaeyOeI0IaeeyEaK3aaSbaaWqaaiabbgdaXaqabaWccqGHflY1cqqG0baDaaGccqqGPaqkcqGHRaWkcqqGbbqqdaWgaaWcbaGaeeOmaidabeaakiabgwSixlabbIcaOiabbgdaXiabgkHiTiabbwgaLnaaCaaaleqabaGaeyOeI0IaeeyEaK3aaSbaaWqaaiabbkdaYaqabaWccqGHflY1cqqG0baDaaGccqqGPaqkaaa@6202@

Recently a 3-parameter bi-exponential model was put forward as a means of calculating the BLC response to MSE [7]. The 3-parameter model was initially developed to model radioactive transformations [13]. Later it was used as pharmacokinetic model [14]. It describes concentrations in the one compartment model as a function of time with first-order invasion and first-order elimination. It requires a BLC value at the start of exercise (BLC_0_). It approximates an increase of lactate (A) in the extra-vascular water space generated by exercise metabolism mainly in working muscles during MSE, which is equivalent to the subsequent decrease to the pre-exercise lactate concentration. It furthermore estimates two velocity constants describing the corresponding kinetics of the appearance (k_1_) and the disappearance (k_2_) of lactate into and out of the blood compartment (Eq.2).

BLC(t)=A⋅k1k2−k1⋅(e−k1⋅t−e−k2⋅t)+BLC0
 MathType@MTEF@5@5@+=feaafiart1ev1aaatCvAUfKttLearuWrP9MDH5MBPbIqV92AaeXatLxBI9gBaebbnrfifHhDYfgasaacPC6xNi=xI8qiVKYPFjYdHaVhbbf9v8qqaqFr0xc9vqFj0dXdbba91qpepeI8k8fiI+fsY=rqGqVepae9pg0db9vqaiVgFr0xfr=xfr=xc9adbaqaaeGacaGaaiaabeqaaeqabiWaaaGcbaacbaGae8NqaiKae8htaWKae83qamKaeiikaGIae8hDaqNaeiykaKIaeyypa0ZaaSaaaeaacqWFbbqqcqGHflY1cqWFRbWAdaWgaaWcbaGaeGymaedabeaaaOqaaiab=TgaRnaaBaaaleaacqaIYaGmaeqaaOGaeyOeI0Iae83AaS2aaSbaaSqaaiabigdaXaqabaaaaOGaeyyXICTaeiikaGIae8xzau2aaWbaaSqabeaacqGHsislcqWFRbWAdaWgaaadbaGaeGymaedabeaaliabgwSixlab=rha0baakiabgkHiTiab=vgaLnaaCaaaleqabaGaeyOeI0Iae83AaS2aaSbaaWqaaiabikdaYaqabaWccqGHflY1cqWF0baDaaGccqGGPaqkcqGHRaWkcqWFcbGqcqWFmbatcqWFdbWqdaWgaaWcbaGaeGimaadabeaaaaa@5AC5@

Differentiation of both models allows for the determination of TBLC_max_, insertion of TBLC_max _in Eq.1 and Eq.2 give the corresponding BLC_max_.

The aims of the present study were 1) to test whether the proposed bi-exponential 3-parameter model sufficiently describes the changes in BLC compared with the previously used 4-parameter model, and 2) to analyse how parameters are changed by the duration of the MSE.

## Methods

Eleven male subjects (mean ± SD age: 24.6 ± 2.3 yrs; height: 182.4 ± 6.8 cm; body mass: 75.1 ± 9.4 kg; body mass index: 22.6 ± 2.5 kg m^-2^; peak oxygen uptake: 4080 ± 228 ml min^-1^) participated in the present study. All subjects were healthy non-smokers, physically active but not specifically trained. None of whom were receiving any pharmacological or specific dietetic treatment. Informed consent was obtained from all subjects after explanation of the nature and risks involved in participation in the experiments, which conformed to internationally accepted policy statements regarding the use of human subjects as approved by the local ethics committee.

Each subject performed two MSE-tests lasting 10 s (MSE10) and 30 s (MSE30) on a mechanically braked cycle ergometer (834 E, Monark) in a randomised order. All tests were carried out at similar times in the morning at least two hours after a light meal. There was a recovery period of one week between testing sessions. The subjects were instructed not to engage in strenuous activity during the day before an exercise test.

In accordance with accepted recommendations for anaerobic performance testing [15], subjects performed a standardised five minutes cycling warm-up at 50 W which included two sprints lasting three seconds performed at the end of the third and the fourth minute as coordinative preparation for MSE10 and MSE30 tests. After a further 10 minutes of rest, the subjects were instructed to pedal as fast as possible. A resistance corresponding to 7.5 % of the body weight was applied after an acceleration phase of three seconds. After termination of each test, the subjects were supervised during a 20 min rest period, where they maintained a seated position.

The BLC was determined from capillary blood samples drawn from the hyperaemic ear lobe. Samples were taken immediately before each test, within 15 s after each test, minute by minute up to the 10^th ^minute, and every second minute up to the 20^th ^minute post-test. Samples were haemolysed and analysed utilizing the enzymatic amperometric method (Ebio Plus, Eppendorf).

Data are reported as mean and standard deviation (mean ± SD). Differences within subjects were analysed using the paired t-test. A multiple nonlinear regression analysis was used for the approximation of the BLC time courses using the bi-exponential 3-parameter model to determine the constants A, k_1 _and k_2_, and also the bi-exponential 4-parameter model with the constants A_1_, A_2_, y_1 _and y_2_. The goodness of the fits of the 3- and the 4-parameter model was compared using the F-test [16]. The interrelationships between selected variables were examined using simple regression analysis. Statistical significance was indicated using an alpha level of 0.05.

## Results

### Performance and BLC

**Figure 1:**
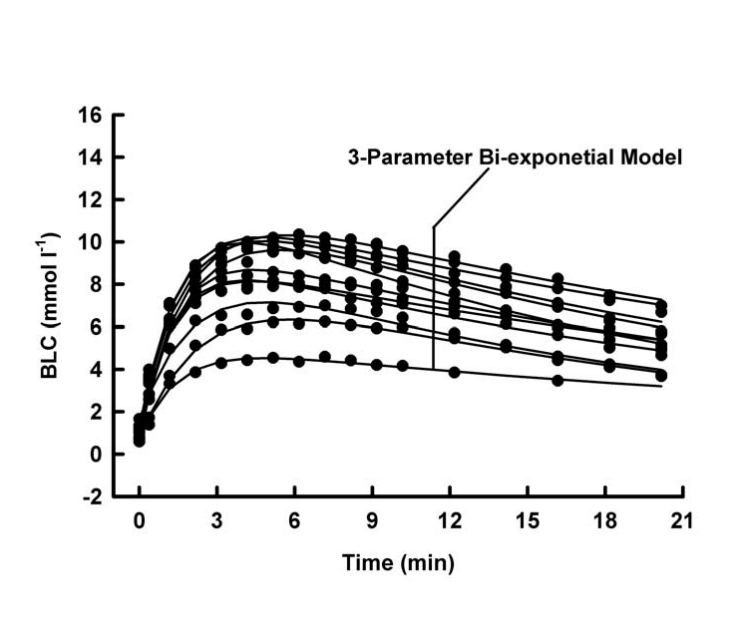
BLC, individual 3-parameter model approximations for MSE10.

**Figure 2:**
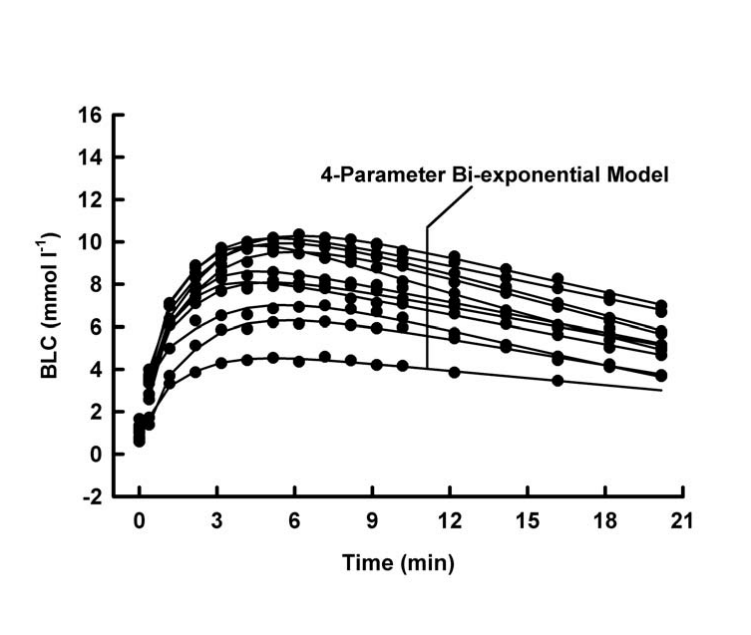
BLC, individual 4-parameter model approximations for MSE10.

**Figure 3:**
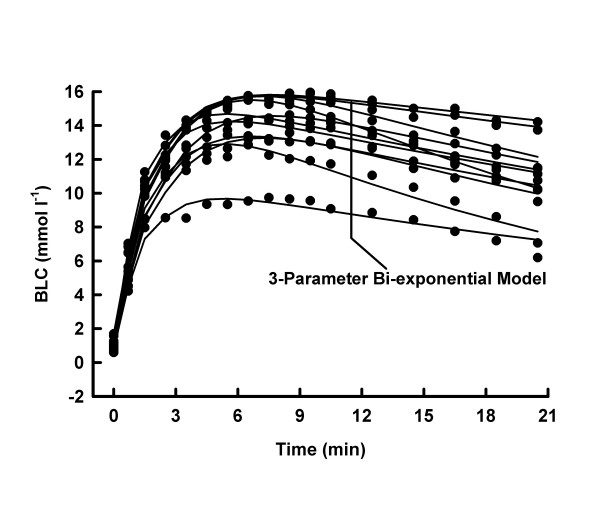
BLC, individual 3-parameter model approximations for MSE30.

**Figure 4:**
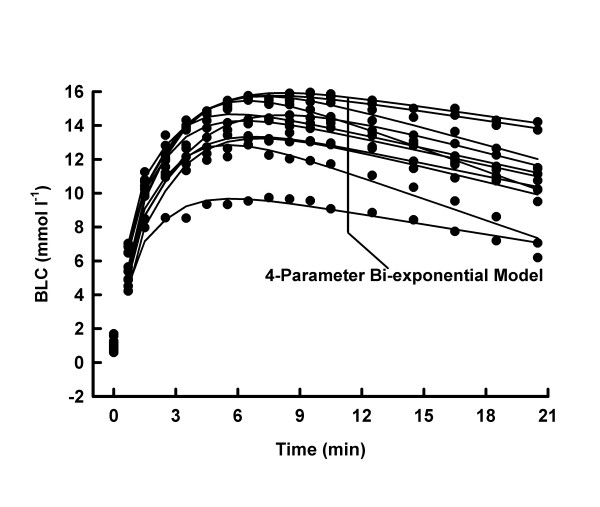
BLC, individual 4-parameter model approximations for MSE30.

Average mechanical power output was higher (10.1 ± 1.0 vs. 8.3 ± 0.7 W kg^-1^, p < 0.05), whilst average mechanical work was lower (100.6 ± 9.8 vs. 248.0 ± 21.5 J kg^-1^, p < 0.05) in MSE10 than in MSE30. At any post MSE time, the BLC was consistently higher (p < 0.05) under MSE30- than under MSE10-conditions (Fig. 1, 2, 3, 4).

### Goodness of fits, parameter estimations, TBLC_max _and BLC_max_

Both models adequately described the BLC response to MSE10 and MSE30, respectively (all R^2 ^≥ 0.9369). The reduction of the number of parameters decreased the goodness of the fit in every MSE10 and in 3 tests under MSE30-conditions (Tab. 1 and 2 and Fig. 1, 2, 3, 4).

**Table 1:** 3-parameter vs. 4-parameter model to describe the BLC-response to MSE10

No	Model	R^2^	SS ((mmol l^-1^)^2^)	A & A_1 _(mmol l^-1^)	k_1 _& y_1_(min^-1^)	A_2 _(mmol l^-1^)	k_2 _& y_2_(min^-1^)	F-test
1	3-Par.	0.9612	1.4722	7.6001	0.4648		0.0665	#
	4-Par.	0.9814	0.4127	6.3018	0.3685	-18.4300	0.0206	
2	3-Par.	0.9882	0.5224	7.4582	0.4197		0.0491	#
	4-Par.	0.9902	0.2586	6.3885	0.5273	-665.7111	0.0003	
3	3-Par.	0.9932	0.7271	11.1438	0.5304		0.0317	#
	4-Par.	0.9975	0.1277	9.0771	0.4665	-403.1144	0.0007	
4	3-Par.	0.9900	0.1645	4.3546	0.6661		0.0318	#
	4-Par.	0.9885	0.0833	3.5289	0.6283	-272.6364	0.0004	
5	3-Par.	0.9934	0.6322	11.2838	0.7024		0.0498	#
	4-Par.	0.9967	0.1685	8.5908	0.6356	-21.6096	0.0209	
6	3-Par.	0.9903	0.7606	9.4807	0.7199		0.0363	#
	4-Par.	0.9924	0.2987	7.3507	0.7579	-1067.4220	0.0002	
7	3-Par.	0.9914	0.8179	10.4780	0.6771		0.0317	#
	4-Par.	0.9935	0.2906	8.0916	0.6162	-961.1428	0.0003	
8	3-Par.	0.9805	1.0944	8.1816	0.7267		0.0348	#
	4-Par.	0.9871	0.3348	6.1414	0.6068	-917.7335	0.0002	
9	3-Par.	0.9871	0.7901	8.4675	0.7458		0.0425	#
	4-Par.	0.9905	0.3065	6.4371	0.8290	-669.8416	0.0003	
10	3-Par.	0.9778	2.0009	10.7703	0.5843		0.0423	#
	4-Par.	0.9928	0.3211	8.3454	0.4849	-838.0681	0.0004	
11	3-Par.	0.9877	1.0402	10.6474	0.4781		0.0464	#
	4-Par.	0.9945	0.2487	8.5783	0.4454	-366.2962	0.0009	

R^2^: coefficient of determination; SS: sum of squared residuals; A: extra-vascular increase of lactate (3-parameter model); A_1_: amplitude of the invasion of lactate into the blood compartment (4-parameter model); k_1 _or y_1_: rate constant of lactate invasion into the blood compartment; A_2_: amplitude of the evasion of lactate out of the blood compartment (4-parameter model); k_2 _or y_2_: rate constant of lactate evasion out of the blood compartment; #: significantly improved goodness of the fit using the 4-parameter model

**Table 2:** 3-parameter vs. 4-parameter model to describe the BLC-response to MSE30

No	Model	R^2^	SS ((mmol l^-1^)^2^)	A & A_1 _(mmol l^-1^)	k_1 _& y_1_(min^-1^)	A_2 _(mmol l^-1^)	k_2 _& y_2_(min^-1^)	F-test
1	3-Par.	0.9813	3.3496	14.9744	0.3805		0.0304	
	4-Par.	0.9613	3.2491	11.5457	0.4533	-1644.1026	0.0002	
2	3-Par.	0.9944	1.2735	16.2805	0.4054		0.0213	#
	4-Par.	0.9946	0.5160	12.5178	0.3504	-733.8497	0.0004	
3	3-Par.	0.9929	1.7610	15.8388	0.5378		0.0098	#
	4-Par.	0.9934	0.5882	11.1687	0.4186	-15.7730	0.0142	
4	3-Par.	0.9754	2.0272	10.0625	0.6528		0.0234	
	4-Par.	0.9369	1.7294	6.4807	0.5995	-1935.3727	0.0001	
5	3-Par.	0.9939	1.5333	18.7569	0.3780		0.0408	
	4-Par.	0.9899	1.2244	14.0299	0.4763	-717.1412	0.0006	
6	3-Par.	0.9711	4.6991	15.1327	0.5009		0.0432	
	4-Par.	0.9560	3.2632	10.3104	0.5295	-3260.3276	0.0001	
7	3-Par.	0.9904	1.7884	15.0838	0.6263		0.0193	#
	4-Par.	0.9831	0.9845	9.8006	0.5342	-1784.7679	0.0001	
8	3-Par.	0.9967	0.6126	14.7640	0.4912		0.0219	
	4-Par.	0.9931	0.5131	10.4533	0.5033	-977.3814	0.0003	
9	3-Par.	0.9937	1.2066	15.2064	0.6656		0.0204	
	4-Par.	0.9880	0.7076	9.4287	0.6027	-1188.8360	0.0002	
10	3-Par.	0.9932	1.6160	15.4396	0.5053		0.0126	
	4-Par.	0.9842	1.4673	10.9240	0.4777	-1177.0599	0.0002	
11	3-Par.	0.9936	1.5344	17.1709	0.4081		0.0270	
	4-Par.	0.9878	1.2859	13.0576	0.4348	-2103.6627	0.0002	

R^2^: coefficient of determination; SS: sum of squared residuals; A: extra-vascular increase of lactate (3-parameter model); A_1_: amplitude of the invasion of lactate into the blood compartment (4-parameter model); k_1 _or y_1_: rate constant of lactate invasion into the blood compartment; A_2_: amplitude of the evasion of lactate out of the blood compartment (4-parameter model); k_2 _or y_2_: rate constant of lactate evasion out of the blood compartment; #: significantly improved goodness of the fit using the 4-parameter model

In both tests, there was no difference between k_1 _of the 3- and y_1 _of the 4-parameter model. Furthermore, all lactate appearance parameters were highly correlated between both models (r > 0.84, p < 0.001). Contrary to the latter in both tests k_2 _was higher (p < 0.05) than y_2 _(Tab. 3 and 4).

**Table 3:** Kinetics of the blood lactate response to given short-term maximal cycling tests based on the bi-exponential 3-parameter model

	MSE10	MSE30
A (mmol l^-1^)	9.1 ± 2.1	15.3 ± 2.1*
K_1 _(min^-1^)	0.610 ± 0.119	0.505 ± 0.107*
K_2 _(min^-1^)	4.21 10^-2 ^± 1.06 10^-2^	2.45 10^-2 ^± 1.04 10^-2^*
BLC_max _(mmol l^-1^)	8.5 ± 1.9^#^	14.1 ± 1.8*
TBLC_max _(min)	4.8 ± 0.6	6.6 ± 1.0*^#^

A: intra-muscular increase of lactate; k_1_: constant of invasion into the blood compartment; k_2_: constant of evasion out of the blood compartment; BLC_max_: highest BLC above the pre-test BLC; TBLC_max_: time of BLC_max_; *: significantly different to MSE10; ^#^: significantly different to 4 parameter model

**Table 4:** Kinetics of the blood lactate response to given short-term maximal cycling tests based on the bi-exponential 4-parameter model

	MSE10	MSE30
A_1 _(mmol l^-1^)	7.1 ± 1.6	10.9 ± 2.0*
y_1 _(min^-1^)	0.579 ± 0.137	0.489 ± 0.076*
A_2 _(mmol l^-1^)	-563.8 ± 370.8	-1412.6 ± 868.8*
y_2 _(min^-1^)	0.41 10^-2 ^± 0.82 10^-2^	0.15 10^-2 ^± 0.42 10^-2^
BLC_max _(mmol l^-1^)	8.4 ± 1.8^#^	14.1 ± 1.8*
TBLC_max _(min)	4.8 ± 0.6	6.1 ± 1.0*^#^

A_1_: amplitude of increase of the BLC; y_1_: constant of invasion into the blood compartment; A_2_: amplitude of decrease of the BLC; y_2_: constant of evasion out of the blood compartment; *: significantly different to MSE10; ^#^: significantly different to 3 parameter model

TBLC_max _in MSE30 and BLC_max _in MSE10 were higher (p < 0.05) using the 3- than the 4-parameter model (Tab. 3 and 4). BLC_max _in MSE10 was also higher (p < 0.05) than the corresponding highest directly measured BLC of 8.4 ± 1.8 mmol l^-1^. All maxima estimated with different models were highly correlated (all r > 0.91, p < 0.001).

### Effect of test duration

**Figure 5:**
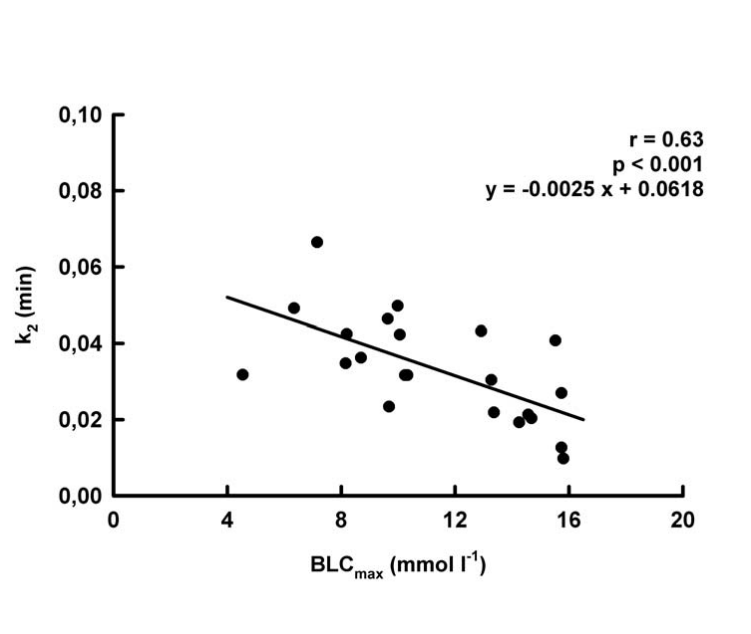
Interrelationship between BLC_max _and k_2_.

The 3-parameter model revealed lower (p < 0.05) values of A, BLC_max _and TBLC_max _in MSE10 than in MSE30 (Tab. 3). k_1 _and k_2 _were higher (p < 0.05) in MSE10 than in MSE30 (Tab. 3). A negative correlation (p < 0.05) between k_2 _and A (r = 0.51; p < 0.005; y = -0.006 x + 0.056) and k_2 _and BLC_max _was found (Fig. 5).

Use of the 4-parameter model showed lower (p < 0.05) values of A_1_, and higher (p < 0.05) levels of A_2 _and y_1 _in MSE10 than in MSE30 (Tab. 4). No significant difference in y_2 _was found between MSE10 and MSE30 (Tab. 4).

## Discussion

Both MSE tests generated normal to high values of power output, work and BLC values for maximal exercise tests of corresponding durations in non-specifically trained healthy male subjects aged 20 to 30 years [6,7,15,17].

Under these conditions, the bi-exponential 3-parameter and the 4-parameter model adequately described the changes in BLC as a cumulative effect of all related factors irrespective of whether any of these factors' specific behaviour may be described adequately using exponential models as well. Reduction of the number of parameters from 4 to 3 did clearly impair the goodness of the fit of the BLC under MSE10- but only to a minor extent under MSE30-conditions.

The observed differences in A and BLC_max _between MSE10 and MSE30 clearly reproduced the frequent observation that after high intensity short term exercise the BLC is not only a result of exercise intensity but also of the duration of the event requiring net lactate production in the extra-vascular compartment. Higher values of A and BLC_max _coincided with an increase of TBLC_max _from approx. 5 min to 7 min. Differences between calculated and directly measured values of BLC_max _of approximately 1 % reflect time intervals of less than ± 1 min of TBLC_max_, which appears practically neglectable under the consideration of usual blood sampling intervals between 1 and 3 min. Both models show that the longer TBLC_max _results from a combined decrease of the rate constants of lactate invasion and lactate evasion.

Neither the 3- nor the 4-parameter model does directly predict the real amount of intra-muscular lactate increase of any specific muscle. MSE10 and MSE30 leg-cycle ergometries are whole-body exercises involving significant contributions from lean tissue masses throughout the entire body [18]. Assuming a muscle mass of 40 % of total body mass and typical dilution factors for total body water and blood water content [7,8,19,20], the A-values estimated for MSE10 and MSE30 do represent a net increase in muscular lactate of 16.9 mmol kg^-1 ^(MSE10) and 27.6 mmol kg^-1 ^(MSE30), equivalent to a rate of net increase in muscular lactate of 1.69 mmol kg^-1 ^s^-1 ^and 0.92 mmol kg^-1 ^s^-1^. These values are well within the range of the magnitude of directly measured intra-muscular lactates and equivalents of approx. 1.18 kJ kg^-1 ^and 1.93 kJ kg^-1 ^of anaerobic lactic energy production found in previous studies that used comparable durations of maximal cycling or electrical stimulation [17,21,22].

Both models provided almost identical results about the invasion of lactate into the blood compartment. This was not the case with respect to the lactate elimination. The 3-parameter model supported previous results that the rate constant decreases if the BLC_max _increases [23-25]. The k_2_-values found in the present experiments using the 3-parameter model represent rate constants of lactate elimination equivalent to BLC half times similar to others determined previously [23-25]. Contrary, the 4-parameter model provided y_2_-estimates equivalent to halftimes of the BLC decrease of approx. 1650 min under MSE10- and approx. 3450 min under MSE30-conditions combined with extreme values of A_2 _(Tab. 1, 2 and 4). These extreme parameter estimations of the 4-parameter model seem to indicate that the use of a relatively short post exercise period combined with the limited number of data points were insufficient for the most successful application of the 4-parameter model, despite an excellent goodness of the fits.

The latter may be indirectly supported by other previous studies, which used the 4-parameter model with recovery periods of up to 120 minutes [10,26-29]. Using the 4-parameter model they estimated A_2 _values not lower than -22.5 mmol l^-1 ^and halftimes of the BLC decrease more or less equivalent with k_2_-values estimated with the 3-parameter model in the present study and others [30] (Tab. 1, 2, 3).

## Conclusion

The 3-parameter model and the 4-parameter model adequately described the changes in BLC under MSE conditions. Reduction of the number of parameters did impair the goodness of the fit of the BLC. However, under the given testing conditions the 3-parameter model seems to provide more realistic parameter estimations with respect to the elimination of lactate from the blood compartment even at relative short periods of blood sampling.

The proposed 3-parameter model seems to provide useful information about the dynamics of lactate in the blood and in the extra-vascular compartment and it is sensitive to changes in test duration.

Increase of the duration of MSE from 10 to 30 s increases BLC_max _and delays TBLC_max _from approx. 5 to approx. 7 min. The latter delay is a combined effect of decreased rate constants of lactate invasion and particularly lactate evasion from the blood compartment.

## Competing interests

The author(s) declare that they have no competing interests.

## Authors' contributions

RB designed and coordinated the study, helped to analyze the data and drafted the manuscript. MDJ conducted the experiments, analyzed the data and helped to draft the manuscript. RML helped to develop the study design and to draft the manuscript. All authors read and approved the final manuscript.

All experiments were conducted at the Institute of Sports Medicine, Free University Berlin.
